# Rickets in Children: An Update

**DOI:** 10.3390/biomedicines9070738

**Published:** 2021-06-27

**Authors:** Cristina Gentile, Francesco Chiarelli

**Affiliations:** Department of Pediatrics, University of Chieti, 66100 Chieti, Italy; cristinagentile9108@gmail.com

**Keywords:** rickets, vitamin D, calcipenic rickets, phosphopenic rickets, FGF-23, X-linked hypophosphatemic rickets, burosumab

## Abstract

Rickets refers to a deficient mineralization of the growth plate cartilage, predominantly affecting longer bones. Despite the fact that preventive measures are available, it is still a common disease worldwide; nutritional rickets, due to vitamin D deficiency or dietary calcium inadequate intake, remains the most common form. Medical history, physical examination, radiologic features and biochemical tests are essential for diagnosis. Although recent studies suggest hypophosphatemia as the leading alteration, rickets is classically divided into two categories: calcipenic rickets and phosphopenic rickets. Knowledge of this categorization and of respective clinical and laboratory features is essential for rapid diagnosis and correct management. The aim of this review is to analyze the epidemiological, pathogenetic, clinical, and therapeutic aspects of the different forms of rickets, describing the novelties on this “long-lived” disease.

## 1. Introduction

Rickets was first described as a specific condition in the mid-seventeenth century [1] and still remains a frequent condition worldwide. Recent reports indicate an increased incidence and prevalence of this disease in industrialized countries; indeed, in the United Kingdom, an overall incidence of 7.5 per 100,000 children younger than five years of age has been reported in the early part of the 21st century [2].

Rickets is a bone disease characterized by abnormal serum calcium (Ca) and phosphate (Pi) levels that may cause the development of abnormalities in chondrocytes’ differentiation and maturation and, consequently, a defective mineralization of the growth plate [3]. It predominantly affects longer bones, leading to poor bone growth and typical rickets bony deformities such as bow-legs and knock-knees [4].

Based on the biochemical predominant abnormalities, rickets was typically classified as calcipenic or phosphopenic, although evidence suggests hypophosphatemia as the leading pathogenetic denominator of all forms [5]. Diagnosis is usually established by medical history, physical examination, biochemical tests, and radiography.

The prevalence of nutritional rickets has substantially declined compared with the prevalence a century ago, but the condition has been re-emerging even in some well-resourced countries [6,7]. Prevention of this form of rickets is possible and includes either supplementation or food fortification with Ca and vitamin D and adequate sunlight exposure. Where prevention has not been effective, treatment with calcium and/or vitamin D supplementation is recommended [8,9,10].

Management of heritable types of rickets associated with defects in vitamin D metabolism or activation involves the administration of vitamin D metabolites [11]. Hypophosphatemic rickets is a heterogeneous group of entities due to renal Pi wasting wherein fibroblast growth factor 23 (FGF23) often plays a major role [12].

X-linked hypophosphatemic rickets (XLHR) is the most common cause of inherited Pi wasting, with an incidence of 3.9 per 100,000 live births [13]. Oral Pi supplementation is usually indicated for FGF23-independent phosphopenic rickets, whereas the conventional treatment of FGF23-dependent types of rickets includes a combination of Pi and activated vitamin D.

An important development has been the introduction of burosumab, a human monoclonal antibody to FGF23, which has been approved for the treatment of XLHR in children one year and older [14].

This review delves into the various aspects of rickets, from pathogenesis to new therapeutic tools, in order to provide a complete and updated picture on this disease and of its appropriate management.

## 2. Epidemiology

Rickets is still a common disease worldwide. Despite a global estimate of rickets, incidence is limited because of the paucity of basic data such as vitamin D dietary intake, especially among children in non-industrialized countries, and recent reports indicate an increase in its prevalence [2,6].

Nutritional rickets remains the most common type globally, representing the most frequent cause of pediatric bone disease in the world [15]. In the United States, incidence estimates of nutritional rickets in the early 2000s was 24.1 per 100,000 compared with 2.2 per 100,000 in the early 1980s [6]. Today, case rate estimates of 2.9–27 per 100,000 individuals have been reported in the United States and Europe [6]. However, nutritional rickets seems to be more common in low-and middle-income countries, especially the Indian subcontinent, Africa, and the Middle East [16]. Studies from Asia continue to report high prevalence rates of nutritional rickets; in rural central Tibet, about 30% of children aged 0–5 years had at least one sign of nutritional rickets on clinical examination [17].

While it is found all over the world, the etiology of nutritional rickets varies geographically. Vitamin D deficiency is the most common cause, especially in temperate countries [16]. However, in settings such as in low- and middle-income countries in Asia and Africa, low dietary Ca intake also plays a crucial role [18].

Although nutritional rickets continues to be an important global health problem, in the last years, the incidence of heritable forms of rickets has increased. This can be explained thanks to the improvement in molecular techniques that has made it possible to identify the genetic alterations of many of these rare forms of rickets. XLHR remains the most common heritable form, with an incidence of 3.9 per 100,000 live births and a prevalence of 1.7 per 100,000 children [19,20,21] and a negative impact on patients’ quality of life [22].

Other heritable forms of rickets are extremely rare and few epidemiological data are available to date.

## 3. Bone Formation and Skeletal Growth

Bone formation and skeletal growth are complex, well-regulated processes in which numerous local and systemic factors play a crucial role.

During childhood, longitudinal growth is determined by the activity of the growth plate cartilage located in the epiphyses of long bones [23]. Chondrocytes contained in the proliferative zone of the growth plates rapidly divide and organize themselves into columns that align parallel to the direction of growth. Part of these will later differentiate into large hypertrophic chondrocytes, forming the hypertrophic zone. These cells undergo apoptosis or trans-differentiation into osteoblasts [24,25,26], leaving empty lacunae that are progressively invaded by blood vessels and osteo-progenitors, which will produce bone matrix using cartilage as a scaffold for the mineralization process. 

Although the complete mechanism is not yet fully understood, recent evidence suggests that bone mineralization starts in matrix vesicles (MVs) derived both from chondrocytes and osteoblasts, classically considered the bone-forming cells [27]. Ca and Pi ions, taken up by specific channels, are expressed on the outer membrane MVs and will crystallize in order to form hydroxyapatite; subsequently hydroxyapatite propagates on collagen fibrils and mineralizes the extracellular matrix.

Tissue non-specific alkaline phosphatase (ALP) is the main factor for Pi production through the degradation of its substrates including pyrophosphate (PPi), adenosine triphosphate (ATP), and the protein-bound form of phosphate [28,29]. However, the role of another phosphatase, PHOSPHO1, has been recently highlighted in mice, although its role in humans still needs to be elucidated [30,31].

Osteocytes, osteoblasts, and osteoclasts are the main cell types orchestrating bone metabolism through a mechanism that is locally and systemically regulated and that are indissolubly linked to Ca and Pi homeostasis in the body. 

Osteocytes are considered the master regulators of bone metabolism thanks to the secretion of both pro- and anti-osteoclastogenic factors such as Receptor Activator of Nuclear factor Kappa-Β Ligand (RANK-L) [32], Macrophage Colony-Stimulating Factor (M-CSF), and Osteoprotegerin (OPG) [33,34]. Moreover, osteocytes are directly involved in bone remodeling through the regulation of sclerostin, which is able to suppress bone formation [35,36], and the synthesis of osteopontin (OPN), a molecule involved in the mineralization process and in hematopoiesis [37]. Moreover, osteocytes appear to be centrally involved in Ca-Pi homeostasis through regulation of the vitamin D signaling pathway [38] and phosphate related-gene expression such as *PHEX* (phosphate-regulating neutral endopeptidase) and *FGF-23* (Fibroblast Growth Factor-23) [39]. On the other hand, osteoclasts are involved in the bone remodeling process; thus they are typically considered the “bone-resorbing” cells. RANKL and M-CSF, predominantly expressed in osteoblasts, represent the two essential factors that can stimulate osteoclast differentiation and activities [40]. Moreover, osteoblasts express OPG, a negative regulator of osteoclast differentiation [41]. Bone-resorbing activity is also directly inhibited by calcitonin (CT), an endogenous calcium regulatory hormone [42,43,44]. Finally, osteoblasts represent the “bone-forming” cells in a process that is regulated by a wide range of cytokines including bone matrix-derived transforming growth factor beta (TGF-β), bone morphogenic protein 2 (BMP-2), BMP-4, BMP-7, and their inhibitors insulin-like growth factor-1 (IGF-1), OPN, and fibroblast growth factors (FGFs) [45].

## 4. Regulation of Mineral Homeostasis

Ca and Pi are the two major components of hydroxyapatite, the crystalline mineral component of bone matrix. Thus, adequate and proportionate availability of Ca and Pi is crucial for proper acquisition and maintenance of bone mass and strength [46]. Moreover, as demonstrated by Sabbagh et al., Pi concentration is directly involved in the regulation of the hypertrophic chondrocytes’ apoptosis pathway in the growth plates [5].

Three major players regulate Ca and Pi homeostasis: calcitriol (1,25-dihydroxyvitamin D), parathyroid hormone (PTH), and FGF23. 

**Figure 1:**
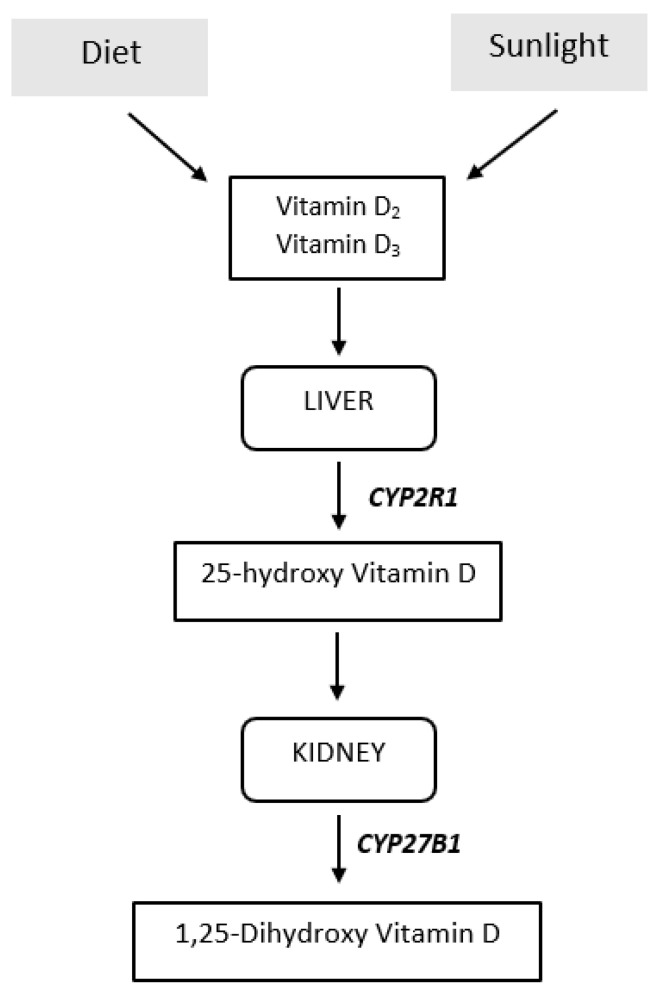
Metabolism of vitamin D.

Calcitriol is the bioactive hormonal form of vitamin D, and derives from the precursors ergocalciferol (vitamin D2) and cholecalciferol (vitamin D3), originating from plant and animal sources, respectively. In the human body, vitamin D3 is synthetized in the skin through ultraviolet B irradiation of the 7-dehydrocholesterol in the epidermis (Figure 1).

Later, vitamin D2 and D3 are transported by the vitamin D binding protein to the liver where they undergo 25-hydroxylation via the P450 enzyme CYP2R1. 25-Hydroxyvitamin D (25(OH)D), the major circulating vitamin D metabolite, will reach the kidneys, where it undergoes a further hydroxylation by 25(OH)D-1α-hydroxylase (CYP27B1) into 1,25-dihydroxyvitamin D (1,25(OH)_2_D) or calcitriol [47]. In its target tissues, calcitriol may determine genomic and non-genomic actions. The first are mediated by the interaction with a high-affinity intracellular nuclear vitamin D receptor (VDR) and a co-receptor, Retinoid x receptor (RXR) [48], whereas non-genomic actions are mediated by the interaction with a plasmatic membrane receptor [49]. One target tissue of calcitriol is the intestine, where this hormone facilitates Ca and Pi absorption [50]. When Ca serum levels are inadequate, calcitriol may also interact with VDR expressed on osteoblasts, promoting osteoclast maturation, and Ca/Pi absorption by skeletal tissue [47]. Calcitriol synthesis, and consequently Ca/Pi serum levels, are increased by hypocalcemia, hypophosphatemia, and PTH while are decreased by calcitriol and FGF23 [4].

The role of PTH in mineral homeostasis has been well characterized. PTH is produced in the parathyroid glands and serves as the primary regulator of serum Ca and, to a lesser extent, of Pi. It acts directly through its target tissues, bone and kidney, to increase the release of Ca from bony stores and to decrease its excretion. Moreover, PTH indirectly increases intestinal Ca/Pi absorption through the stimulation of renal 25(OH)D-1α-hydroxylase. In the kidney, PTH also induces a rapid removal of the NaPi-2a protein from the proximal renal apical membrane and promotes its degradation by lysosomes. Thus, the net effect of PTH is to increase plasmatic Ca levels and also decrease Pi [51].

FGF-23, mainly produced by osteoblasts and osteocytes, represents the principal regulator in the maintenance of serum Pi levels [52]. Despite its expression in several extra-osseous tissues [53], recent studies have highlighted the role of a single-pass transmembrane protein named αKlotho in signal transduction through FGFr, identifying it as being mainly responsible for FGFr specificity [54]. Thus, only organ and tissues that express both FGFr and Klotho can be targets for the physiological action of FGF23. Kidneys are the main target for FGF23; indeed, FGFr activation results in the removal of NaPi 2a/2c from the apical side of proximal tubule cells and in the suppression of 25(OH)D-1α-hydroxylase, with a consequent increase in Pi urinary loss and a decrease in calcitriol synthesis and intestinal Ca/Pi absorption [55]. The regulation of FGF23 is complex and involves multiple components on the bone–parathyroid–kidney axis [56]. High Pi serum concentrations and calcitriol increases FGF23 levels [57,58,59,60,61,62,63], while low Ca levels have been shown to decrease its levels, subsequently removing FGF23 suppression of calcitriol [64,65]. The effects of PTH on FGF23 are not entirely clear. Although animal models and in vitro studies indicate that PTH directly stimulates FGF23 production [66,67,68,69,70], results concerning human studies are conflicting [71,72]. In addition, post-translational regulatory mechanisms including PHEX and Dentin Matrix Acidic Phosphoprotein 1 (DMP-1 ) have been highlighted in bone [56].

**Figure 2:**
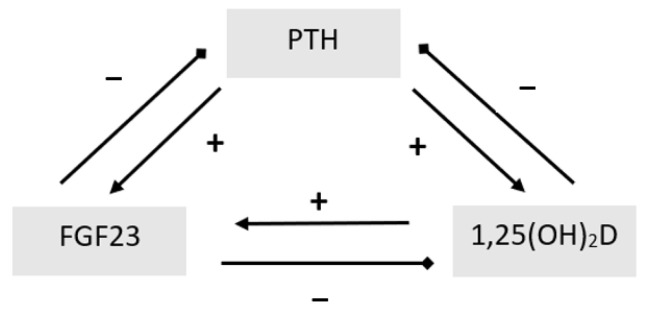
PTH/FGF23/1,25(OH)_2_D interaction.

Hence, there is a complex interaction between calcitriol, PTH, and FGF23 (Figure 2). Understanding these interactions is essential for the comprehension, diagnosis, and management of rickets.

## 5. Classification and Pathogenesis

Traditionally, rickets may be classified into two major groups: calcipenic and phosphopenic [4].

Calcipenic rickets is primarily caused by inadequate Ca intake, which is most commonly due to vitamin D deficiency. Less frequently, calcipenic rickets arises from defects in the metabolic pathway of vitamin D or from target tissue resistance to calcitriol. Reduced intestinal Ca absorption leads to the activation of the parathyroid–bone axis in order to preserve blood Ca concentrations. The results, mediated by PTH, are the activation of bone resorption, a decrease in renal calcium loss and, finally, a decrease in tubular phosphate resorption and hyphosphatemia [73].

Phosphopenic rickets results from deficient/impaired intestinal Pi absorption or abnormal renal excretion of Pi, which can be isolated or a part of generalized tubular dysfunction (i.e., Fanconi syndrome) [12,73].

**Scheme 1:**
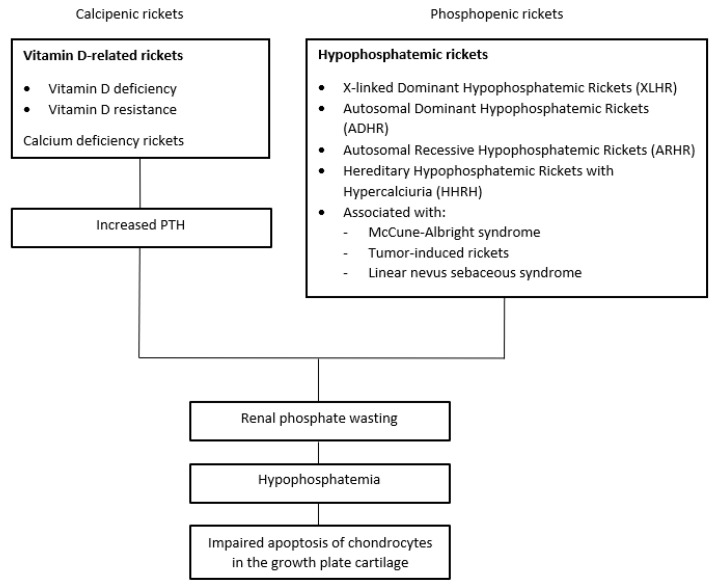
Clinical features.

Hypophosphatemia is directly responsible for the reduced apoptosis of the growth plate’s hypertrophic chondrocytes and, consequently, for the development of clinical and radiological rachitic changes [74]. Thus, despite the different mechanisms, hypophosphatemia can be considered the common denominator of both calcipenic and phosphopenic rickets [5,74,75] (Scheme 1).

## 6. Clinical Features

Bone deformities are the hallmark of rickets. They typically appear before the age of 18 months with the maximum frequency reported between the ages of 4 and 12 months. Rapid bone growth areas including costochondral junctions and long bone epiphyses are predominantly affected.

Bone deformity types depend upon child weight-bearing patterns of the limbs [3]. Forearm deformities are common in crawling infants, whereas bow legs (genu varum) or knocks knees (genu valgum) develop more frequently in toddlers [73]. Florid signs of rickets are rare in adolescents, which usually manifest with vague symptoms such as headache and pain in the lower limbs. Severe rickets may also present with “rachitic rosary” due to the expansion of the costo-chondral junctions. Growth retardation, frontal bossing of the skull, craniotabes (soft skull bones), delayed tooth eruption, and widened fontanelles have also been reported [73,76]; moreover, an increased susceptibility to fractures has been shown [77].

Extra skeletal manifestations can also occur that include proximal muscle weakness, hypotonia, irritability [65], hypo calcemic seizures, tetany, laryngospasm and, rarely, cardiomyopathy [78,79]. These conditions are typical of vitamin D deficiency rickets and are less frequently observed in patients with hypophosphatemic rickets, especially XLHR. In contrast, patients with hypophosphatemic rickets can develop spontaneous dental abscesses and enamel defects [4]. Craniosynostosis, defined as the premature closure of cranial sutures, and Chiari I malformation have been reported as presenting signs in patients with XLHR [80,81].

## 7. Radiographic Features

Although rickets affects all metaphysis, radiologic alterations are best visualized on radiographs of rapidly growing bones growth plates (i.e., knees, distal ulna, or ankles metaphysis) [3]. These changes also tend to be more marked in toddlers rather than in adolescents. 

The earliest radiological signs of rickets include the loss of the crisp line, produced by the zone of provisional calcification at the interface of the epiphyseal growth plate. In the advanced stages of rickets, this zone may appear frayed (“brush like”) or concave (“cup shaped”). The metaphyseal area also becomes wider than normal. Epiphyseal bone centers may appear small, osteopenic, and ill-defined or their appearance may be delayed [82]. In addition, in calcipenic rickets, radiological signs of secondary hyperparathyroidism can be evident including generalized osteopenia, subperiosteal bone resorption, and periosteal reaction along the diaphysis [73]. In several cases, pathological fractures and Looser’s zones may be noted [83]. In contrast, radiological signs of hypophosphatemic rickets are usually less marked and a thickening of cortices can be found [73]. To facilitate evaluation of the radiographic severity of rickets and of the response to treatment, a 10-point scoring method, the Rickets Severity Score (RSS), has been defined, based on the degree of metaphyseal fraying, concavity, and proportion of the growth plate involved [84].

Although x-ray has been universally approved for rickets diagnosis, several studies are trying to define the role of other imaging techniques. Recently, it has been demonstrated that MRI is a valuable tool to depict rachitic changes in cartilage, especially in XLHR [85]. However, further studies are needed.

## 8. Laboratory Findings

If rickets is suspected, a thorough biochemical evaluation is recommended for a prompt diagnosis. Laboratory findings can change according to the type of rickets and stage of the disease. Salient features of different types of rickets are shown in Table 1.

**Scheme 2:**
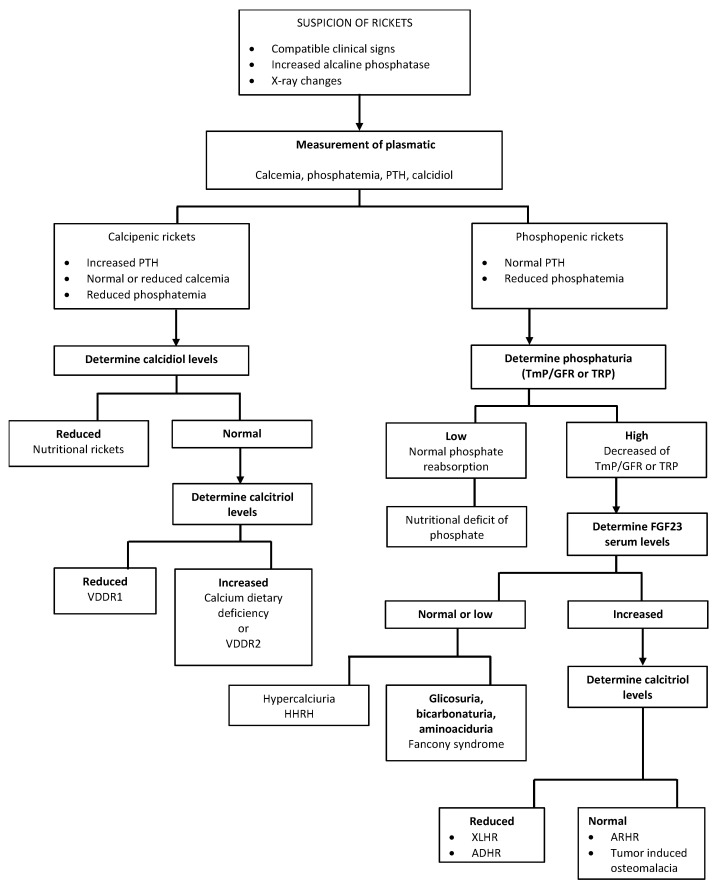
VDDR1: Vitamin D Dependent Rickets type 1; VDDR2: Vitamin D Dependent Rickets type 2; TmPi/GFR: tubular maximum reabsorption of phosphate (TmP) to GFR; TRP: tubular reabsorption of phosphate; XLHR: X-Linked Hypophosphatemic Rickets; ARHR: Autosomal Recessive Hypophosphatemic Rickets; ADHR: Autosomal Dominant Hypophosphatemic Rickets; HHRH: Hereditary Hypophosphatemic Rickets with Hypercalciuria.

Blood ALP measurement is pivotal when rickets is suspected. Elevated ALP activity can confirm the diagnosis in patients with bone deformities and epiphyseal enlargement. Moreover, its levels reflect disease activity, representing an important monitoring tool for treatment [3]. Recently, a biphasic association of ALP serum levels with the severity of genu varum in children with vitamin D deficient rickets has also been described [86]. Scheme 2 (Scheme 2) gives an example of biochemical algorithmic approach to a child with suspected rickets.

## 9. Calcipenic Rickets

### 9.1. Nutritional Rickets

Nutritional rickets refers to rickets due to vitamin D deficiency and/or low Ca intake. Despite a significant decrease in the incidence during the twentieth century, vitamin D deficiency remains one of the most prevalent nutritional deficiencies [87], especially in some ethnic minorities of industrialized countries and in Asiatic population [88,89,90,91,92]. Table 2 shows the classification of the severity of vitamin D deficiency [8].

In the literature, several cut-offs have been proposed for vitamin D deficiency. A recent Global Consensus on the prevention and management of nutritional rickets has defined deficiency as vitamin D level lower than 30 ng/mL [8]. The risk of rickets increases with the decrease in vitamin D serum concentrations and it is particularly high below 10 ng/mL vitamin D levels, even in the presence of an adequate Ca intake [93].

The diagnosis of nutritional rickets is based on clinical, radiological, and biochemical evaluation.

Environmental risk factors can predispose children to develop nutritional rickets and should be taken into account when rickets is suspected (Table 3).

Infancy is an age group quite vulnerable to vitamin D deficiencies. During pregnancy, fetal vitamin D stores are exclusively dependent on maternal vitamin D status and increasing scientific evidence suggests that hypovitaminosis D in the mother has an impact on both maternal and child health [94]. Moreover, in breastfed newborns, the risk of vitamin D deficiency is more elevated than in formula-fed neonates (especially preterms), because of the mineral fortification of these products [95]. On the other hand, preterm infant, especially those with birth weight less than 1000 g, are at high risk for rickets because of deprivation of mineral, 80% of which occurs in the third trimester [96].

Clinical signs of rickets typically develop toward the end of the first year of life and in the course of the second year of life; subsequently becoming subtle [47]. Both skeletal and extraskeletal manifestation may be present and include bone deformities, frontal bossing, bone pain, rachitic rosary, muscle weakness, respiratory infections, and hypocalcemia-related symptoms [3,73,78,79]. Diffuse osteopenia of the long bones may be the earliest radiological sign on x-rays; fraying and cupping deformities of metaphysis, pathological fractures, and Looser’s zones may be observed in severe cases [3,73,82,83]. If nutritional rickets is suspected, biochemical evaluation is also recommended [10]. Serum 25(OH)D, PTH, ALP, Ca, and Pi level dosage are helpful to confirm the diagnosis and also to differentiate nutritional rickets to other causes of rickets (see Table 1) [8,97,98,99]. Most children with vitamin D deficiency rickets have serum 25(OH)D concentrations less than 10 ng/mL and usually less than 5 ng/mL [73]. Calcium and phosphate serum levels may be normal or low while ALP and PTH concentrations are usually elevated.

Vitamin D administration represents the mainstay of nutritional rickets treatment [9,10]. Some regimens have been proposed; however, treatment usually consists of an intensive phase followed by a maintenance phase [8,9,100]. The Global Consensus on nutritional rickets recommends the administration of 2000 UI/day of cholecalciferol in patients aged less than one year, 3000–6000 UI/day in patients aged one to 12 years, and 6000 UI/day in patients older than 12 years for three months, followed by 400 to 600 UI/day in the maintenance phase [8]. In addition, Ca supplementation is important, in order to prevent “hungry-bone syndrome” [100]. Munns et al. recommended oral Ca administration of 500 mg/day regardless of age or weight, reserving intravenous Ca therapy in the presence of acute, symptomatic hypocalcemia [8].

Prevention of vitamin D deficiency can be practiced through adequate sun exposure, vitamin D supplementation, fortification of dietary intake, and adequate Ca intake. Regular vitamin D supplementation is recommended for infants from birth until 12 months of age (400 UI/day). For older children, 600 IU/day of vitamin D through diet or supplementation is advised [8,97,101].

### 9.2. Calcium Deficiency Rickets

Calcium deficiency rickets, frequent in South Africa, Nigeria, and Bangladesh is primarily due to inadequate dietary Ca intake [102]. Clinical signs usually present after 18 months of age. Serum concentrations of 25(OH)D may be normal or slightly reduced, but often PTH and 1,25(OH)_2_D elevated concentrations are associated. Casupplements alone lead to the healing of rickets.

### 9.3. Vitamin D Dependent Rickets

The term “vitamin D dependent rickets” (VDDR) refers to a group of genetic disorders characterized by early-onset rickets due to the inability to maintain adequate concentrations of active forms of vitamin D or a failure in activated vitamin D post-receptor response [11].

Currently VDDR may be divided into three categories. VDDR type 1 (VDDR1) arises from a failure in complete activation of calcitriol, due to inability to generate either 25(OH)D (VDDR1b) or 1,25(OH)_2_D (VDDR1a). The second category (VDDR2) is characterized by calcitriol’s resistance due to mutations in the VDR (VDDR2a) or the alteration of VDR–DNA interaction (VDDR2b). VDDR3 results from an abnormal inactivation of vitamin D metabolites.

Because hypophosphatemia is the common denominator of all forms of rickets [5], the management of the VDDR aims to maintain Ca serum levels in mid-normal range, ensuring a normal serum level of PTH and, consequently a correction of hypophosphatemia. Patients should be regularly monitored with serum and urinary biochemistries, renal ultrasounds, and radiographs [11].

#### 9.3.1. Vitamin D Dependent Rickets Type 1a (VDDR1a)

VDDR1a results from biallelic mutations in the *CYP27B1* gene on chromosome 12q13.3 that encodes 25-hydroxyvitamin D_3_ 1α-hydroxylase. Therefore, patients with VDDR1a are unable to convert 25(OH)D to 1,25(OH)_2_D. About 60 different *CYP27B1* mutations have been identified [103]. The disease is inherited in an autosomal-recessive manner [73]. Children present between two and 24 months with hypotonia, irritability, tetany, or seizures. If the condition is diagnosed later, fractures, bone deformities, and impaired growth can occur [11,75]. Biochemical studies show hypocalcemia, hypophosphatemia, and elevated ALP and PTH serum levels. Plasmatic concentrations of calcitriol are low or undetectable, while 25(OH)D levels are normal or increased [11]. Because of 25-hydroxyvitamin D_3_ 1α-hydroxylase block, treatment with calcitriol or 1α-hydroxyvitamin D (alfacalcidol) is required. Ergocalciferol, cholecalciferol, or 25(OH)D (calcifediol) can be used [11]. The maintenance dose is usually lower than that initially, but treatment must be continued indefinitely. Adequate intake of dietary calcium should be maintained (30–75 mg/kg per day of elemental Ca) [101]. A close monitoring of potential side effects of hypercalcemia, hypercalciuria, and nephrocalcinosis secondary to calcitriol therapy is necessary in these children [101].

#### 9.3.2. Vitamin D Dependent Rickets Type 1b (VDDR1b)

VDDR1b is a very rare condition due to loss-of-function mutations in *CYP2R1*, with decreased expression of 25-hydroxylase. Although a new missense mutation, p.K242N, has recently been described [104], the most common *CYP2R1* mutation is p.L99P. Some studies have suggested a gene dose effect on the rickets phenotype, with a more severe outcome in homozygous patients. In contrast, heterozygous patients for the p.L99P mutation showed only a modest reduction in circulating 25(OH)D concentration and a less severe bone disease [11]. VDDR1b clinical features are similar to that of VDDR1a; nevertheless, the disease’s phenotype appears to improve with age, probably due to the acquisition of vitamin D independent mechanism(s) for the intestinal absorption of Ca [105]. Similar to VDDR1a, patients show hypocalcemia, hypophosphatemia, and elevated ALP and PTH serum levels with low concentrations of 25(OH)D. The administration of calcifediol, which bypasses the enzymatic defect, and supplemental Ca restores vitamin D and mineral homeostasis [106]. Treatment will be required lifelong.

#### 9.3.3. Vitamin D Dependent Rickets Type 2a (VDDR2a)

VDDR2a, or hereditary 1,25(OH)_2_D resistant rickets (HVDRR), is a rare form of rickets due to biallelic loss-of-function mutations in the VDR encoding gene on chromosome 12q13.11, causing bone resistance to vitamin D. Children with VDDR2a present early in life, between two and eight months. Clinical features are very similar to other forms of VDDR, but in two-thirds of cases, alopecia is present due to a failure in vitamin D activity on hair follicle cycling [107]. Alopecia may be partial or complete and appears to be associated with a worst disease’s phenotype, with very early onset of hypocalcemia and poor response to therapy [101,108]. Laboratory tests show low serum levels of Ca and Pi and elevated serum concentrations of calcitriol (50–1000 pg/mL). This finding differentiates VDDR2 from VDDR1.

To date, no effective treatment is available [79,109] and the most accredited strategy is to saturate the normal receptors through mega-doses of calcitriol and Ca. During the first few months of life, oral or intravenous Ca therapy can be required to restore normocalcemia and reverse secondary hyperparathyroidism [11]. As affected infants grow, Ca and vitamin D balance changes and additional therapy with high-dose vitamin D therapy may be necessary [11]. Maintenance treatment depends on calciferol therapy response. Some patients, especially those with alopecia, fail to respond to maximal dose of calciferol and then require prolonged intravenous Ca administration [110,111].

#### 9.3.4. Vitamin D Dependent Rickets Type 2b (VDDR2b)

VDDR2b is a rare disorder closely related to VDDR2a. To date, no specific gene has been identified as the cause of VDDR2b. It seems to be caused by the overexpression of a nuclear protein that attenuates transcription of the DNA response element that binds RXR-VDR heterodimers [11]. Despite the different pathogenesis, VDDR2b resembles VDDR2a and the treatment is similar.

#### 9.3.5. Vitamin D Dependent Rickets Type 3 (VDDR3)

VDDR3 is a rare autosomal dominant form of rickets due to gain-of-function missense mutation in the *CYP3A4* gene that leads to an increased and rapid inactivation of vitamin D metabolites [112]. Despite few cases have been reported, VDDR3 appears to be very similar to VDDR1, with bone deformities as the most frequent manifestation [112]. Affected children present with detectable serum concentration of colecalciferol, but low levels of 25(OH)D and 1,25(OH)_2_D, which require very high doses of calcitriol or colecalciferol (50,000 UI daily) to normalize.

## 10. Phosphopenic Rickets

### 10.1. X-Linked Dominant Hypophosphatemic Rickets (XLHR)

XLHR is the most common form of hypophosphatemic rickets with an incidence of 1:20,000 [113]. It results from the inactivating mutations in the *PHEX* gene, expressed in osteocytes and odontoblasts. These determine an increased synthesis and secretion of FGF23, which regulates renal tubular resorption of Pi [114]. Increased FGF23 levels result in renal wasting of Pi at the proximal tubule level and reduced 1-α-hydroxylation of 25-OH vitamin D [115]. Since the identification of the first *PHEX* mutation in 1995, at least over 300 mutations have been registered and, recently, a novel *PHEX* mutation in exon 22 has been described. However, how *PHEX* gene mutations increase FGF23 remains unknown [116].

XLHR manifests as a spectrum of disorders, from hypophosphatemia alone to short stature. Frontal bossing is one of the initial clinical signs, appearing as early as six months of age. As the children start walking, other signs become evident including coxa vara, genu valgum, or varum. Stunted growth may be a revealing sign in up to 14% of cases; therefore any leg bowing whether or not associated with poor statural growth should be investigated [117]. Tooth abscesses or facial cellulitis on apparently non-carious teeth are common and suggest poor dentin mineralization [118]. Unlike vitamin D deficiency, craniotabes, rachitic rosary, and fractures are rare [4]. Finally, recent studies have shown higher incidence of craniovertebral abnormalities, especially craniosynostosis and Chiari type 1 malformation [80,81].

Biochemical hallmarks of XLHR include hypophosphatemia, increased ALP, and elevated serum levels of FGF23 [119,120].

A diagnosis of XLHR should be considered in the presence of clinical and/or radiological signs of rickets, impaired growth velocity, and hypophosphatemia associated with Pi renal wasting without vitamin D or Ca deficiency. Where possible, diagnoses should be confirmed by genetic testing. Any first-generation family member of a patient with XLHR should be investigated for XLHR [13]. However, approximately one-third of reported patients have a negative family history for XLHR; in these patients, a mutational analysis for the *PHEX* gene is also recommended [121,122]. If genetic analysis is not available, elevated plasma levels of FGF23 and/or a positive family history for XHLR can be used to support the diagnosis [13].

Conventional treatment of XLHR includes phosphate and active vitamin D (calcitriol or alfacalcidol) oral supplementation. Recommended doses vary from 20–60 mg/kg body weight daily for phosphate supplements, and 20–30 ng/kg for calcitriol or 30–50 ng/kg for alfacalcidol [13]. Conventional treatment should be continued until the completion of growth. Adverse effects including nephrocalcinosis and secondary hyperpathyroidism can occur. Nephrocalcinosis, which happens subsequently to urinary calcium loss, is reported in 30–70% of patients with XLHR; therefore, periodic urinary Ca monitoring is recommended [13]. Secondary hyperparathyroidism is the consequence of prolonged stimulation of the parathyroids by FGF23 and Pi supplementation, and most frequently occurs in patients not treated with calcitriol or alfacalcidol. Thus, to prevent it, PTH levels during conventional treatment should be maintained within 10–65 pg/mL [13]. Adjuvant therapy with cinacalcet, a calcimimetic agent, can be considered in patients with persistent secondary hyperparathyroidism. In a small pediatric study, cinacalcet has been demonstrated to be effective in reducing serum PTH and FGF23 levels [123]. However, cinacalcet treatment is not licensed for this indication because of its potentially severe side effects such as hypocalcemia and increased QT interval [124]. Therefore, parathyroidectomy should be considered in patients with tertiary hypercalcemic hyperparathyroidism [13].

An important novelty in the treatment of XLHR was the introduction of burosumab (KRN23), a recombinant human monoclonal IgG1 antibody that targets FGF23, restoring renal Napi co-transporter [14]. Evidence-based European guidelines recommend burosumab treatment in children older than one year and in adolescents with growing skeletons in the presence of: (a) radiographic evidence of overt bone disease; (b) disease refractory to conventional therapy; (c) complication related to conventional therapy; and (d) noncompliance to conventional therapy. Recommended starting dose is 0.4 mg/kg subcutaneously every two weeks [13]. Burosumab is generally a well-tolerated drug; injection site reactions are the most frequently reported side effects. Other possible adverse reactions include headache, vomiting, pyrexia, and extremities pain. Despite its benefits, various aspects of burosumab in XLHR therapy are yet to be explored and further studies are needed.

In addition to antibodies against FGF23, other biological therapies are under development and could be used in the near future [125]. In a minority of patients, orthopedic, odontoiatric, and neurosurgical measures may be necessary [14].

### 10.2. Autosomal Dominant Hypophosphatemic Rickets (ADHR)

ADHR is caused by activating missense mutations in *FGF23* that make the protein resistant to the cleavage by the FGF23-targeting converting enzyme, leading to an increased expression of FGF23 and phosphaturia [126]. Clinical findings are similar to those in XLHR, especially in childhood onset, whereas bone pain, weakness and pseudofractures are more common in adolescence [127]. In addition, several studies have shown an association between iron deficiency ad severe disease manifestations [128]. Treatment of ADHR is similar to that of XLHR and includes Pi and calcitriol administration. Iron therapy should be prescribed if iron deficiency is present [129].

### 10.3. Autosomal Recessive Hypophosphatemic Rickets (ARHR)

Several inactivating mutations can cause ARHR, determining different phenotypes of the disease.

ARHR type 1 results from the loss of function mutations in *DMP1*, leading to increase in FGF23 expression and impaired skeletal mineralization [130,131].

ARHR type 2 has recently been described. It occurs because of a loss of function mutations in ectonucleotide pyrophosphate/phosphodiesterase 1 gene (*ENPP1*), which regulates matrix vesicle pathway and pyrophosphate-mediated bone mineralization [132]. The results are impaired bone mineralization, idiopathic infantile arterial calcification, ossification of the posterior longitudinal ligament of the spine, and insulin resistance [133,134]. Clinical, laboratory, and radiological findings of patients with ARHR are similar to those of XLHR patients.

### 10.4. Hereditary Hypophosphatemic Rickets with Hypercalciuria (HHRH)

Hereditary hypophosphatemic rickets with hypercalciuria results from the loss of function mutations in the *SLC34A3* gene that encodes NaPi-2c, with consequent tubular Pi wasting and hypophosphatemia [135,136,137]. An increased number of novel mutations in the *SLC34A3* gene are described every day: indeed, five novel gene mutations have recently been reported [138]. Chronic Pi urinary loss leads to suppression of FGF23, calcitriol, and PTH secretion, with a consequent increase in Ca absorption in the gut and into secondary hypercalciuria, the hallmark of HHRH. Hypercalciuria predisposes to nephrolithiasis and progressive renal failure [139]. Bone pain, muscle weakness, and pseudofractures in childhood are common presenting features in patients with homozygous or compound heterozygous *SLC34A3* mutations. Dental abnormalities are not usually reported. In contrast, heterozygous carriers present later in life with idiopatic hypercalciuria and mild hypophosphatemia and/or elevated calcitriol serum levels. Bone disease is generally absent in these patients [140]. Supplementation with oral Pi may heal rickets and represents the mainstay of HHRH [141,142]. In contrast with other forms of hypophosphatemic rickets, calcitriol administration should be avoided because it increases the risk of developing nephrocalcinosis/recurrent nephrolithiasis [73].

### 10.5. Hypophosphatemic Rickets Associated with McCune-Albright Syndrome/Fibrous Dysplasia (MAS/FD), Linear Nevus Sebaceous Syndrome and Tumor Induced Rickets

In approximately 50% of cases, hypophosphatemic rickets may be associated with McCune-Albright Syndrome/Fibrous Dysplasia (MAS/FD), a group of skeletal disorders characterized by the replacement of normal bone by fibrous connective tissue in single or multiple sites, respectively. In these, hypophosphatemic rickets is caused by excessive FGF23 production by dysplastic osteogenic cells in fibrous lesions; hence treatment is analogue to that of XLHR [73].

Although very rare, increased FGF23 production may be associated with mesenchymal tumors [143] or Linear Nevus Sebaceous Syndrome (LNSS), leading to hypophosphatemia and rickets [144].

## 11. Conclusions

Rickets is a disorder of growing children that arises from defective mineralization of the growth plate cartilage. Although typically classified into calcipenic and phosphopenic rickets, hypophosphatemia is the common pathogenic denominator of all type of rickets.

Nutritional rickets still remains an important global problem; dietary, cultural, environmental, and genetic factors contribute to its frequency. However, every day, new mutations causing heritable types of rickets are discovered, leading to a better knowledge of the underlying mechanisms of these disorders and to new therapeutic opportunities.

## Figures and Tables

**Table 1:** Salient features of different types of rickets.

Type	Calcium	Phosphate	Alkaline Phosphatase	PTH	25(OH)D	1,25(OH)_2_D
**Calcipenic Rickets**						
**Vitamin D deficiency**	L or N	L or N	H or VH	H	L	variable
**VDDR1**	L	L or N	VH	H	N	L
**VDDR2**	L	L or N	VH	H	N	H
**Phosphopenic rickets**						
**XLHR**	N	L	H	N or H	N	L or N
**ADHR**	N	L	H	N	N	L
**ARHR**	N	L	H	N	N	L
**HHRH**	N	L	H	L or N	N	H

VDDR1: Vitamin D-dependent Rickets type 1; VDDR2: Vitamin D-dependent Rickets type 2; XLHR: X-linked Hypophosphatemic Rickets; ADHR: Autosomal Dominant Hypophosphatemic Rickets; ARHR: Autosomal Recessive Hypophosphatemic Rickets; HHRH: Hereditary Hypophosphatemic Rickets with Hypercalciuria; 25(OH)D: 25-hydroxy vitamin D; 1,25(OH)_2_D: 1,25-dihydroxy vitamin D; L: low; N: normal; H: high; VH: very high.

**Table 2:** Severity of 25(OH) vitamin D deficiency.

Vitamin D Status	ng/mL
Deficiency	<30
Insufficiency	30–50
Adequate	>50
Toxicity	>250

**Table 3:** Environmental risk factors for vitamin D deficiency rickets.

Environmental Risk Factors for Vitamin D Deficiency Rickets
Dark skin pigmentation
Living at a high latitude during winter or spring with lack of ultraviolet B radiation exposure as a consequence
Restricted sun exposure, for example, due to cultural skin covering or pollution
Lack of vitamin D supplementation during infancy
Prolonged exclusive breastfeeding
Maternal vitamin D deficiency
